# Effects of vitamin K supplementation on reproductive performance and bone metabolism-related biochemical markers in lactation sows

**DOI:** 10.5713/ab.23.0092

**Published:** 2023-05-04

**Authors:** Huakai Wang, Yu Zhang, Yongxi Ma

**Affiliations:** 1 State Key Laboratory of Animal Nutrition, College of Animal Science and Technology, China Agricultural University, Beijing 100193, China

**Keywords:** Bone Metabolism-related Biochemical Marker, Reproductive Performance, Sow, Vitamin K

## Abstract

**Objective:**

This study was conducted to evaluate the effects of vitamin K (VK) supplementation on reproductive performance and bone metabolism-related biochemical markers in sows.

**Methods:**

Twenty-four Large White×Landrace sows (mean parity 4.04) were randomly assigned to two dietary treatments (NC diet, a basal diet with 0.5 mg/kg of VK_3_; VK diet, a basal diet with 5 mg/kg of VK_3_) with twelve replicates per treatment and one sow per replicate according to parity. The experiment started on day 107 of gestation and lasted until day 21 of lactation (weaning).

**Results:**

We observed that there were no differences (p>0.05) in average daily feed intake, backfat loss of sows, live piglet number at birth and weaning, average birth weight, average weaning weight, and average daily gain of piglets between two treatments. The apparent total tract digestibility of phosphorus was increased (p<0.05) in VK sows compared with NC sows. The serum bone alkaline phosphatase, osteocalcin, type I procollagen amino-terminal peptide, and type I procollagen carboxyl-terminal peptide on day of farrowing were higher (p<0.05) in VK sows than in NC sows. The serum phosphorus, parathyroid hormone, tartrate-resistant acid phosphatase, and tumor necrosis factor-alpha on day of weaning were lower (p<0.05) in VK sows compared with NC sows.

**Conclusion:**

Therefore, the overall results suggested that increasing dietary VK_3_ (0.5 to 5 mg/kg) during lactation improved the apparent total tract digestibility of phosphorus and serum bone metabolism biochemical markers in sows.

## INTRODUCTION

Sow longevity is a factor affecting the efficiency and profit of pig farming; however, approximately 70% of sows are removed before they attain their peak production due to reasons such as reproductive failure and leg problems [1]. Lameness in sows is one of many problems that damage animal health and causes economical loss to producers. It is reported that locomotion disability resulted in approximately 6% to 40% (mean 10%) of breeding sows removal [2,3]. Moreover, there are higher piglet losses (27.7% in lame sows vs 12.4% in non-lame sows) and fewer litters (<3.0 litters in lame sows vs. 4.5 litters in non-lame sows) in lame sows than healthy sows [4], along with fewer pigs born, piglet mortality rate. Lameness was affected by a variety of environmental factors including genetics [5], diet composition [6], growth rate [7], and mechanical stress [6]. Thus, it is critical to study the bone nutrition of sows and reduce the elimination rate of sows caused by lameness.

Sows under modern breeding conditions, with an average of more than 2.2 litters per year, are at different stages of reproduction throughout their life. Rapid fetal growth and development in late pregnancy and postpartum lactation require a large amount of calcium (Ca) and phosphorus (P), which may be the physiological causes of bone malnutrition and metabolic diseases in sows. However, excessive supplementation of Ca, P and other minerals and vitamin D3 did not improve the bone nutrition of sows, which suggests that the bone malnutrition of sows did not originate from mineral deficiency, but was due to the inability to deposit calcium and phosphorus in the bones [8]. Vitamin K (VK) promotes the carboxylation of osteocalcin (OC), which binds with calcium ions to form a mature mineralized matrix [9]. Furthermore, previous studies showed that VK could activate a variety of glutamate proteins including OC and promote osteoblast differentiation by pathways such as steroid and xenobiotic receptor, proteinkinase A, and bone morphogenetic protein [10–12]. However, the potential regulatory effects of VK on bone metabolism in sows have not been studied before. It is difficult to precisely distinguish a mildly lame sow from a healthy sow, while bone metabolism biochemical markers can reflect the state of bone turnover with sensitivity and specificity. The purpose of this study was to determine the effects of VK supplementation on reproductive performance and bone metabolism-related biochemical markers in sows, to explore the effects of VK on bone health of sows.

## MATERIALS AND METHODS

All animal experiments are carried out in accordance with the requirements of experimental animal welfare and animal experiment ethics of China Agricultural University (Beijing, China) (No. AW70203202-1-1).

### Experimental design and diets

On day 107 of gestation, 24 sows (Large White×Landrace, mean parity 4.04) were selected and randomly allotted to two dietary treatments (n = 12 sows/treatment) based on their parity. The dietary treatments were VK_3_ contents of 0.5 or 5 mg/kg (NC diet vs VK diet) throughout the experiment. Different contents of VK_3_ were prepared into premix and then mixed with other feed materials. The diets were formulated to meet or exceed the recommended requirements by the National Research Council [13] (Table 1).

From day 107 of gestation to day 21 of lactation (weaning), sows were placed in individual farrowing crates (2.0 m×3.0 m). Temperature in the farrowing room was maintained at a minimum of 20°C and supplemental heat was provided for piglets using heat lamps. The sows were fed approximately 2.0 kg/d of experimental diet from day 107 of gestation until the day of farrowing and then the amount of diet was increased daily by 1.0 kg until ad libitum feeding. The feed was provided three times daily and water was provided *ad libitum* during the whole lactating period. The litter size was standardized to 12–13 piglets and piglets received iron dextran solution (1 mL per piglet), tails were docked, ears were notched, and teeth were cut within 24 h after farrowing.

### Sampling and measurements

Individual sows were scanned for backfat thickness two times (farrowing and weaning) to determine backfat thickness changes. Backfat thickness was measured at P2 position (6 cm from the midline at the head of the last rib) with an ultrasonic device (Piglog105; SFK Technology A/S, Herlev, Denmark). Feed was weighed daily to calculate the average daily feed intake (ADFI) during the lactating period. Piglets were weighed at birth and on weaning to calculate average daily gain (ADG).

From days 19 to 21 of lactation, 100 g of faeces from sows was collected and dried at 65°C for 72 h. Diets and faeces were ground to pass a 1-mm screen (40 mesh) before analysis and analyzed for dry matter (DM, method 934.01), Ca (method 968.08), and P (method 985.01) [14]. Apparent total tract digestibility (ATTD) of DM, Ca, and P were determined.

On day of farrowing and day of weaning, blood samples from sows were collected via jugular vena puncture into vacutainer tubes (Becton Dickinson Vacutainer Systems, Franklin Lakes, NJ, USA) and then centrifuged at 3,000×g for 15 min to get the serum samples. The concentrations of Ca and P in the serum samples were analyzed with an automatic biochemical analyzer (TBA-120FR; Toshiba Co., Ltd., Tokyo, Japan) using arsenazo III and phosphomolybdate methods. The levels of serum parathormone (PTH), bone alkaline phosphatase (BALP), OC, type I procollagen amino-terminal peptide (PINP), type I procollagen carboxyl-terminal peptide (PICP), osteoprotegerin (OPG), tartrate-resistant acid phosphatase (TRACP), type I collagen carboxy-terminal peptide (CTX-I), type I collagen amino-terminal peptide (NTX), interleukin-1β (IL-1β), interleukin-6 (IL-6), tumor necrosis factor-alpha (TNF-α), transforming growth factor-beta (TGF-β), and insulin-like growth factor (IGF) were analyzed using ELISA kits (Nanjing Jiancheng Bioengineering Institute, Nanjing, China) according to the instructions. Serum calcitonin and growth hormone concentrations were analyzed using a radioimmunoassay system (XH6080; Xi 'an Nuclear Instrument Factory, Xi 'an, China).

### Statistical analysis

All data were analyzed using SAS 9.4 (SAS Inst. Inc., Cary, NC, USA). Each sow (litter) or piglet was considered as the experimental unit. Models included treatment as the fixed effect and replicate as the random effect. The t-test procedure of SAS 9.4 was used to detect the differences between NC and VK treatments. Results were presented as mean±standard error of mean. Significant differences were considered at p< 0.05.

## RESULTS

### Reproductive performance

No differences were observed (p>0.05) in ADFI, backfat loss of sows, survival number at birth and weaning, average birth weight, average weaning weight, and ADG of piglets between two treatments (Table 2).

### Nutrient digestibility

The ATTD of P was increased (p<0.05) in VK sows compared with NC sows (Table 3). There were no effects of dietary treatments on ATTD of DM and Ca.

### Bone metabolism-related biochemical marker

There were no differences (p>0.05) in serum bone metabolism-related biochemical markers of sows on day of farrowing (Table 4). As shown in Table 5, the serum P, PTH, TRACP, and TNF-α on day of weaning were lower (p<0.05) in VK sows compared with NC sows. The serum BALP, OC, PINP, and PICP on day of farrowing were higher (p<0.05) in VK sows than in NC sows.

## DISCUSSION

Mammals can synthesize VK_2_ through their gut flora [15]. It is considered that microbial synthesis is sufficient to meet the requirements of animals [16]. However, sows rely mainly on feed for VK due to little possibility of contact with feces under modern production conditions. The NRC [13] has established requirements as 0.5 mg menadione (a commercial form of VK_3_) per kg feed for all production phases. The recent review by Yang et al. on the strategies for the supplementation of vitamins and trace minerals in pig production showed that the average concentration of VK_3_ in lactating diet increases to 4.47 mg/kg feed, almost nine times greater than that recommended by the NRC [17]. Vitamin K deficiency causes defective blood coagulation and anemia [16], which may affect animal growth performance. In the current study, the reproductive performance of sows was not affected by the additive amount of dietary VK_3_, indicating that the NRC recommended amount could meet the reproductive requirement of sows. However, it is reported that VK deficiency has a more pronounced effect on bone than on blood coagulation, and VK requirements for maintaining bone health was higher than the dietary reference values [18]. Thus, we further examined the ATTD of Ca and P and bone metabolism-related biochemical marker in sows.

Calcium and P account for 1.5% to 2.2% and ~1% of total body weight, most of this being present in bone in the form of hydroxyapatite which contributes to the maintaining of bone health [19]. Sows have a high requirement for Ca and P due to fetal growth and milk secretion. Therefore, inadequate intake or reduced bioavailability of Ca and P causes Ca and P deficiency in sows, which can even lead to lameness and postpartum paralysis, thus shortening the reproductive life of sows. Sows fed the VK diet had increased the ATTD of P and reduced serum P concentration compared with those fed the NC diet in our study, indicating the beneficial effects of VK on bone phosphorus deposit. Parathyroid hormone is synthesized and secreted by the main cells of the parathyroid gland, which plays an important role in maintaining calcium and phosphorus balance and regulating bone metabolism. Previous research found that PTH could upregulate the expression of NF-κB receptor activator ligand (RANKL) in osteoclasts through the PTH receptor signaling pathway, thereby inducing bone resorption [20]. Qu et al [21] also reported that the concentration of serum PTH is inversely associated with the bone mineral density of several bones. Our findings demonstrated that VK could reduce the concentration of serum PTH, which indicated that VK may improve bone health through organismal P metabolism.

Bone mass is maintained by a balance of osteoblast-mediated bone formation and osteoclast-mediated bone resorption [22]. The turnover between formation and resorption causes the release of bone-derived molecules that can be measured in blood and urine [23]. Biochemical markers of bone formation include i) BALP, which is secreted from bone; ii) OC, which is a VK-dependent calcium-binding protein; iii) PINP and PICP, which are cleaved during the processing of type I collagen; and iv) OPG, which is an osteoclastogenesis inhibitory factor [23]. Moreover, biochemical markers of bone resorption include TRACP, which is an osteoclast-derived enzyme, and CTX-I and NTX, which are proteolytic fragments generated by cathepsin K cleavage of type I collagen [23]. In the present study, the VK diet increased the concentrations of BALP, OC, PINP, and PICP while inhibiting the concentration of TRACP compared with the NC diet in serum of sows, indicating that VK might improve bone health, as shown by the improvement of bone metabolism-related biochemical markers. Moreover, we detected the hormones and cytokines associated with bone metabolism. Tumor necrosis factor-alpha, a proinflammatory cytokine, had proven to stimulate osteoclast proliferation and differentiation and suppress osteoblast activity at stages of differentiation [24]. Our results showed that VK diet decreased the level of TNF-α compared with the NC diet, while the mechanism needs further studied.

In conclusion, the present study indicates that higher dietary VK content improved ATTD of P and bone metabolism-related biochemical markers during lactation in sows without affecting their reproductive performance, more research is needed to investigate the possible mechanism of VK_3_ on phosphorus and serum bone metabolism in sows.

## Figures and Tables

**Table 1:** Ingredients and chemical composition of the basal diets (as-fed basis)

Items	Contents
Ingredients (%, as-fed basis)	
Corn	64.42
Soybean	28.00
Wheat bran	3.00
Soybean oil	1.50
Dicalcium phosphate	1.38
Limestone	0.90
Salt	0.30
Vitamin and mineral premix^1)^	0.50
Calculated nutrient level (%)	
Digestible energy (MJ/kg as fed)	15.41
Crude protein	17.94
Lysine	0.86
Methionine	0.27
Threonine	0.58
Tryptophan	0.16
Calcium	0.79
Available phosphorus	0.39

1) Provided per kg of diet: vitamin A, 12,000 IU; vitamin D_3_, 1200 IU; vitamin E, 24 IU; thiamin, 2 mg; riboflavin, 6 mg; pyridoxine, 4 mg; vitamin B_12_, 24 μg; niacin, 30 mg; pantothenic acid, 20 mg; folic acid, 3.6 mg; biotin, 1 mg; choline chloride, 0.4 g; 96 mg of Fe (as FeSO_4_·H_2_O); 8 mg of Cu (as CuSO_4_·5H_2_O); 120 mg of Zn (as ZnSO_4_·H_2_O); 40 mg of Mn (MnSO_4_·H_2_O)0.56 mg of I (as Ca(IO_3_)_2_); 0.4 mg of Se (sodium selenite); 0.5 mg vitamin K_3_ for NC treatment or 5 mg vitamin K_3_ for VK treatment.

**Table 2:** Effects of vitamin K_3_ supplementation on reproductive performance in sows

Items	NC^1)^	VK^1)^	SEM	p-value
Sows				
ADFI (kg)	5.83	5.73	0.18	0.80
Backfat loss	1.33	1.40	0.19	0.87
Piglets				
Number of piglets at birth	12.83	12.18	0.49	0.64
Number of piglets at weaning	11.67	11.00	0.21	0.12
Initial litter weight (kg)	17.89	17.64	0.067	0.85
Litter size at weaning (kg)	64.20	63.23	1.77	0.80
Average birth weight (kg)	1.42	1.46	0.04	0.63
Average weaning weight (kg)	5.50	5.70	0.12	0.40
ADG (g)	193.96	202.03	4.56	0.40

SEM, standard error of the mean; ADFI, average daily feed intake; ADG, average daily gain.

1) NC, 0.5 mg vitamin K_3_/kg feed; VK, 5 mg vitamin K_3_/kg feed.

**Table 3:** Effects of vitamin K_3_ supplementation on nutrient digestibility (%) in sows

Items	NC^1)^	VK^1)^	SEM	p-value
Dry matter	87.39	86.17	0.63	0.36
Ca	36.84	35.51	1.55	0.70
P	27.86	39.80	0.84	<0.05

SEM, standard error of the mean; Ca, calcium; P, phosphorus.

1) NC, 0.5 mg vitamin K_3_/kg feed; VK, 5 mg vitamin K_3_/kg feed.

**Table 4:** Effects of vitamin K_3_ supplementation on bone metabolism-related biochemical markers in sows on day of farrowing

Items	NC^1)^	VK^1)^	SEM	p-value
Ca (mmol/L)	2.28	2.29	0.04	0.91
P (mmol/L)	2.09	2.08	0.05	0.89
PTH (pg/mL)	5.84	5.73	0.45	0.95
CT (ng/mL)	52.16	47.70	3.80	0.70
BALP (ng/mL)	13.73	11.91	0.86	0.35
OC (ng/mL)	38.13	39.22	1.51	0.74
PINP (ng/mL)	260.83	242.67	20.65	0.69
PICP (ng/mL)	693.63	668.37	46.40	0.80
OPG (ng/L)	264.92	270.46	13.68	0.87
TRACP (U/L)	10.03	11.42	0.75	0.38
CTX-I (ng/mL)	2.35	2.31	0.17	0.92
NTX (nmol/L)	1,384.32	1,417.67	121.9	0.90
GH (ng/mL)	0.72	0.69	0.07	0.86
IL-1β (pg/mL)	4.75	4.18	0.37	0.48
IL-6 (pg/mL)	8.20	8.48	0.62	0.84
TNF-α (pg/mL)	3.40	3.34	0.30	0.92
TGF-β (pg/mL)	2,665.83	2,540.92	113.90	0.62
IGF (ng/mL)	9.36	10.61	0.66	0.36

SEM, standard error of the mean; Ca, calcium; P, phosphorus; PTH, parathyroid hormone; CT, calcitonin; BALP, bone alkaline phosphatase; OC, osteocalcin; PINP, type I procollagen amino-terminal peptide; PICP, type I procollagen carboxyl-terminal peptide; OPG, osteoprotegerin; TRACP, tartrate-resistant acid phosphatase; CTX-I, type I collagen carboxy-terminal peptide; NTX, type I collagen amino-terminal peptide; GH, growth hormone; IL-1β, interleukin-1β; IL-6, interleukin-6; TNF-α, tumor necrosis factor-alpha; TGF-β, transforming growth factor-beta; IGF, insulin-like growth factor.

1) NC, 0.5 mg vitamin K_3_/kg feed; VK, 5 mg vitamin K_3_/kg feed.

**Table 5:** Effects of vitamin K_3_ supplementation on bone metabolism-related biochemical markers in sows on day of weaning

Items	NC^1)^	VK^1)^	SEM	p-value
Ca (mmol/L)	2.35	2.32	0.02	0.65
P (mmol/L)	1.57	1.31	0.05	<0.05
PTH (pg/mL)	8.59	6.42	0.50	<0.05
CT (ng/mL)	39.24	36.65	3.79	0.52
BALP (ng/mL)	11.99	17.42	1.39	<0.05
OC (ng/mL)	27.56	37.61	2.31	<0.05
PINP (ng/mL)	192.26	260.51	16.32	<0.05
PICP (ng/mL)	530.52	772.62	55.17	<0.05
OPG (ng/L)	271.45	274.88	19.80	0.93
TRACP (U/L)	10.88	7.73	0.56	<0.05
CTX-I (ng/mL)	2.09	1.78	0.14	0.27
NTX (nmol/L)	2,023.30	1,637.20	159.61	0.22
GH (ng/mL)	0.61	0.52	0.05	0.45
IL-1β (pg/mL)	1.48	1.06	0.15	0.17
IL-6 (pg/mL)	5.33	6.31	0.41	0.26
TNF-α (pg/mL)	4.24	2.66	0.27	<0.05
TGF-β (pg/mL)	2,796.51	2,960.13	153.08	0.62
IGF (ng/mL)	8.08	7.72	0.73	0.82

SEM, standard error of the mean; Ca, calcium; P, phosphorus; PTH, parathyroid hormone; CT, calcitonin; BALP, bone alkaline phosphatase; OC, osteocalcin; PINP, type I procollagen amino-terminal peptide; PICP, type I procollagen carboxyl-terminal peptide; OPG, osteoprotegerin; TRACP, tartrate-resistant acid phosphatase; CTX-I, type I collagen carboxy-terminal peptide; NTX, type I collagen amino-terminal peptide; GH, growth hormone; IL-1β, interleukin-1β; IL-6, interleukin-6; TNF-α, tumor necrosis factor-alpha; TGF-β, transforming growth factor-beta; IGF, insulin-like growth factor.

1) NC, 0.5 mg vitamin K_3_/kg feed; VK, 5 mg vitamin K_3_/kg feed.

## References

[1] Engblom L, Stalder K, Lundeheim N (2011). Premature removal and mortality of commercial sows. Book of abstracts of the 62nd annual meeting of the European Federation of Animal Science.

[2] Anil SS, Anil L, Deen J (2009). Effect of lameness on sow longevity. J Am Vet Med Assoc.

[3] McNeil B, Calderon Diaz J, Bruns C (2018). Determining the time required to detect induced sow lameness using an embedded microcomputer-based force plate system. Am J Anim Vet Sci.

[4] Pluym LM, Van Nuffel A, Van Weyenberg S, Maes D (2013). Prevalence of lameness and claw lesions during different stages in the reproductive cycle of sows and the impact on reproduction results. Animal.

[5] Aasmundstad T, Kongsro J, Wetten M, Dolvik NI, Vangen O (2013). Osteochondrosis in pigs diagnosed with computed tomography: heritabilities and genetic correlations to weight gain in specific age intervals. Animal.

[6] Fabà L, Gasa J, Tokach MD, Varella E, Solà-Oriol D (2018). Effects of supplementing organic microminerals and methionine during the rearing phase of replacement gilts on lameness, growth, and body composition. J Anim Sci.

[7] de Koning DB, van Grevenhof EM, Laurenssen BF, van Weeren PR, Hazeleger W, Kemp B (2013). The influence of dietary restriction before and after 10 weeks of age on osteochondrosis in growing gilts. J Anim Sci.

[8] Morris JG (2008). Vitamins in animal and human nutrition.

[9] Tsugawa N, Shiraki M (2020). Vitamin K nutrition and bone health. Nutrients.

[10] Tabb MM, Sun A, Zhou C (2003). Vitamin K2 regulation of bone homeostasis is mediated by the steroid and xenobiotic receptor SXR. J Biol Chem.

[11] Ichikawa T, Horie-Inoue K, Ikeda K, Blumberg B, Inoue S (2007). Vitamin K2 induces phosphorylation of protein kinase A and expression of novel target genes in osteoblastic cells. J Mol Endocrinol.

[12] Katsuyama H, Saijoh K, Otsuki T, Tomita M, Fukunaga M, Sunami S (2007). Menaquinone-7 regulates gene expression in osteoblastic MC3T3E1 cells. Int J Mol Med.

[13] NRC (2012). Nutrient requirements of swine (11th revised ed.).

[14] AOAC (2012). Official methods of analysis.

[15] Karl JP, Fu X, Wang X (2015). Fecal menaquinone profiles of overweight adults are associated with gut microbiota composition during a gut microbiota-targeted dietary intervention. Am J Clin Nutr.

[16] Litta G (2012). Optimum vitamin nutrition.

[17] Yang P, Wang HK, Li LX, Ma YX (2021). The strategies for the supplementation of vitamins and trace minerals in pig production: surveying major producers in China. Anim Biosci.

[18] Tsugawa N, Uenishi K, Ishida H (2012). A novel method based on curvature analysis for estimating the dietary vitamin K requirement in adolescents. Clin Nutr.

[19] Watanabe O, Hara H, Kasai T (2000). Effect of a phosphorylated guar gum hydrolysate on increased calcium solubilization and the promotion of calcium absorption in rats. Biosci Biotechnol Biochem.

[20] Ben-awadh AN, Delgado-Calle J, Tu X (2014). Parathyroid hormone receptor signaling induces bone resorption in the adult skeleton by directly regulating the RANKL gene in osteocytes. Endocrinology.

[21] Qu Z, Yang F, Hong J (2000). Parathyroid Hormone and Bone Mineral Density: A Mendelian Randomization Study. J Clin Endocrinol Metab.

[22] Zaidi M (2007). Skeletal remodeling in health and disease. Nat Med.

[23] Voorzanger-Rousselot N, Garnero P (2007). Biochemical markers in oncology. Part I: molecular basis. Part II: clinical uses. Cancer Treat Rev.

[24] Wang T, He C (2020). TNF-α and IL-6: the link between immune and bone system. Curr Drug Targets.

